# Applications and Challenges of Machine Learning to Enable Realistic Cellular Simulations

**DOI:** 10.3389/fphy.2019.00247

**Published:** 2020-01-21

**Authors:** Ritvik Vasan, Meagan P. Rowan, Christopher T. Lee, Gregory R. Johnson, Padmini Rangamani, Michael Holst

**Affiliations:** 1 Department of Mechanical and Aerospace Engineering, University of California San Diego, La Jolla, CA, United States,; 2 Department of Bioengineering, University of California San Diego, La Jolla, CA, United States,; 3 Allen Institute of Cell Science, Seattle, WA, United States,; 4 Department of Mathematics, University of California San Diego, La Jolla, CA, United States,; 5 Department of Physics, University of California San Diego, La Jolla, CA, United States

**Keywords:** machine learning, cellular structures, segmentation, reconstruction, meshing, simulation

## Abstract

In this perspective, we examine three key aspects of an end-to-end pipeline for realistic cellular simulations: reconstruction and segmentation of cellular structures; generation of cellular structures; and mesh generation, simulation, and data analysis. We highlight some of the relevant prior work in these distinct but overlapping areas, with a particular emphasis on current use of machine learning technologies, as well as on future opportunities.

## 1. INTRODUCTION

Machine learning (ML) approaches, including both traditional and deep learning methods, are revolutionizing biology. Owing to major advances in experimental and computational methodologies, the amount of data available for training is rapidly increasing. The timely convergence of data availability, computational capability, and new algorithms is a boon for biophysical modeling of subcellular and cellular scale processes such as biochemical signal transduction and mechanics [1]. To date, many simulations are performed using idealized geometries that allow for the use of commonly used techniques and software [2–6]. This is historically due to the lack of high-resolution structural data as well as the theoretical and computational challenges for simulations in realistic cellular shapes, due to the complexity of generating high-quality, high-resolution meshes for simulation, and the need to develop specialized fast numerical solvers that can be used with very large unstructured mesh representations of the physical domain.

As biophysical methods have improved, the complexity of our mathematical and computational models is steadily increasing [7–9]. A major frontier for physics-based study of cellular processes will be to simulate biological processes in realistic cell shapes derived from various structural determination modalities [10, 11]. For biological problems ranging from cognition to cancer, it has long been understood that cell shapes are often correlated with mechanism [12–16]. Despite such clear correlations, there remain gaps in our understanding of how cellular ultrastructure contributes to cellular processes and the feedback between cellular structure and function. Challenges such as the diffraction limit of light and difficulties in manipulation of intracellular ultrastructure constrain the potential scope of what can be achieved experiments. Much like the partnership between biophysics and molecular dynamics simulations have enabled the modeling of invisible protein motions to shed insights on experimental observations, simulations of cellular processes can also aid in the validation and generation of hypothesis currently inaccessible by experimental methods. Recently, we and others have shown that, for example, cell shape and localization of proteins can impact cell signaling [4, 5, 12, 17, 18].

**FIGURE 1 |:**
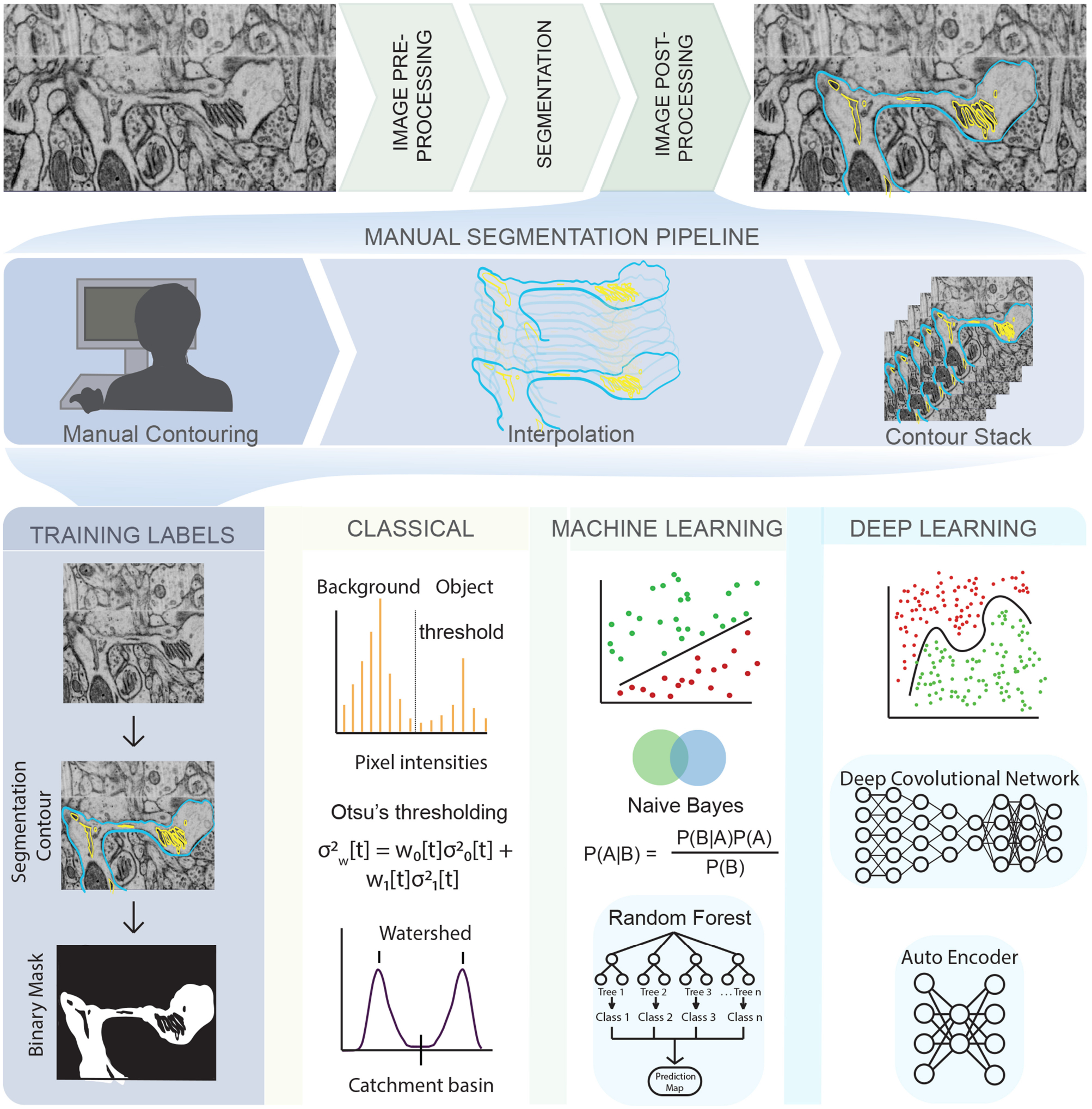
An illustration of the complex pipeline needed to go from imaging data to a segmented mesh, with various opportunities for emerging techniques in machine learning shown throughout the pipeline. **(Top row)** EM images obtained from Wu et al. [24] of dendritic spines from mouse brain tissue. **(Middle row)** Manual tracing or contouring, interpolation, and stacking of contours is extremely time consuming, prone to error, and relies of human judgement. **(Bottom row)** On the other hand, development of training labels and different learning techniques can reduce both time and error, bridging the gap between biological data and simulations. Classical algorithms like Otsu’s thresholding and watershed are widely used and convenient but prone to error. Traditional machine learning algorithms like Random Forest and Naive Bayes are less prone to error and easy to use but require manual painting/interaction. Deep learning algorithms are highly effective and require no manual interaction but are limited by large training sets and compute resources. The list of techniques described is representative only, and not exhaustive.

The major bottleneck for the widespread use of cell scale simulations with realistic geometries is not the availability of structural data. Indeed, there exist many three-dimensional imaging modalities such as confocal microscopy, multiphoton microscopy, super-resolution fluorescence and electron tomography [19, 20]. For example, automation of modalities such as Serial Block-Face Scanning Electron Microscopy is already enabling the production of data at rapid rates. The bottleneck lies in the fact that much of the data generated from these imaging modalities need to be manually curated before it can be used for physics-based simulations. This current *status quo* of manually processing and curating these datasets for simulations is a major obstacle to our progress. In order to bridge the gap between abundance of cellular ultrastructure data generated by 3D electron microscopy (EM) techniques and simulations in these realistic geometries, innovations in machine learning (ML) methods will be necessary to reduce the time it takes to go from structural datasets to initial models. There are already many similar efforts at the organ/tissue and connectomics length scales [21–23]. In this work, we summarize the main steps necessary to construct simulations with realistic cellular geometries (Figure 1) and highlight where innovation in ML efforts are needed and will have significant impacts. We further discuss some of the challenges and limitations in the existing methods, setting the stage for new innovations for ML in physics-based cellular simulations.

## 2. SOURCES OF ERROR IN IMAGING MODALITIES

Images generated by the various microscopy modalities must undergo pre-processing to correct for errors such as uneven illumination or background noise [25, 26]. The choice of suitable algorithms for error correction depends on multiple factors, some of which are listed here—the length scale of the experiment being conducted, scalability and reproducibility of the experiment, optical resolution of the microscope, sensitivity of the detector, specificity of the staining procedure, imaging mode (2D, 3D, 3D time-series), imaging modality (fluorescence, EM, ET etc.,) and other imaging artifacts like electronic noise, lens astigmatism, mechanical tilting/vibration, sample temperature, and discontinuous staining [25–28]. These sources of error are an important consideration for model implementation further downstream [6].

Electron tomography (ET) remains one of the most popular methods of cell imaging for modeling purposes [29–31], as it retains the highest resolution of all the 3D cell imaging techniques [26] by reconstructing a 3D object from a series of 2D images collected at different tilt angles [32]. However, images from ET also have a low signal to noise ratio (SNR) and have anisotropic resolution (for example, 1 nm resolution in x, y and 10 nm resolution in z) [25]. This is partly because biological samples can withstand only a limited dose of electron beam radiation (SNR is proportional to the square root of the electron beam current) before the specimen is damaged [33]. Other sources of error such as misalignment of projections and missing wedges from an incomplete tilt angular range can significantly affect the quality of the reconstruction. To work with data such as these, image processing steps are required for high resolution 3D reconstruction [25, 34]. Commonly used software packages for image processing such as IMOD [35] and TomoJ [36] use reconstruction algorithms such as Weighted Backprojection (WBP) and Simultaneous Iterative Reconstruction Technique (SIRT). While these have been very effective at reconstruction, sources of error can still accumulate, leading to further manual adjustment [37].

## 3. APPLICATIONS OF ML FOR THE SEGMENTATION AND RECONSTRUCTION OF CELLULAR STRUCTURES

Given a noisy 3D reconstruction, how can we segment cellular structures of interest? One approach is to employ manual segmentation tools applied to 3D tomograms such as XVOXTRACE [32, 38], and more generally, manual contouring, interpolation, and contour stacking (Figure 1). The advantage of such methods is that the human eye performs exceptionally well at detecting objects in an image [27, 39]. Consequently, semi-manual and manual segmentation are widely adopted, favoring accuracy over efficiency. However, such methods can be extremely tedious and not always reproducible. Alternatively, numerous semi-automatic segmentation algorithms such as interpolation, watershed, thresholding, and clustering are available as plugins in software packages like IMOD [35] and ImageJ [40] (Figure 1, classical). However, the accuracy of open platform algorithms is debatable [41] because of two main reasons—(i) Even with a “perfect” ET reconstruction (no tilt misalignment, no missing wedge, no noise), the application of filtering algorithms like Gaussian blur or non-linear anisotropic diffusion (NAD) [42] can cause artifacts that lead to misclassifications, rendering the image unsuitable for downstream quantitative simulations and analysis. (ii) Segmentation workflows are often designed for a specific structure and/or imaging modality, limiting their generalizability and applicability.

Annual cell segmentation challenges are evidence of the demand for automatic segmentation [43, 44], with many of its past winners responding with ML-based programs [45, 46]. Training labels for ML techniques requires a relatively small percentage (as small as 10%) of manually segmented labels, allowing for very large data sets to be processed significantly faster than previously implemented semi-automatic segmentation methods. The most successful teams utilized ML techniques such as random forest classifiers, support vector machines, or a combination of these to get segmentations comparable or often even better than their human counterparts [43–46] (Figure 1, machine learning). These techniques function by imputing several image features such as noise reduction, and texture and edge detection filters [47]. These filters are then used to train a classification algorithm in an interactive manner, achieving better classification accuracy at the cost of increased training time compared to the direct application of a filter. However, because the algorithm is interactive, it still requires manual input and both the training time and accuracy can depend on the user.

More recently, deep learning-based ML algorithms (Figure 1, deep learning), and more specifically, convolutional neural networks (CNNs) have surged in popularity due to the success of AlexNet in the ImageNet classification challenge [48]. CNNs are complex learnable non-linear functions that do not require the imputation of data-specific features. Indeed, CNNs learn the feature mapping directly from the image. The U-Net convolutional neural network architecture [46] further generalized deep learning, winning the ISBI neuronal structure segmentation challenge in 2015 with a quicker speed and with fewer training images. It functions by using the feature mapping imputed by a CNN to map the classification vector back into a segmented image. Such is the achievement of the U-Net that its variants are now the state-of-the-art in tasks like calling genetic variation from gene-sequencing data [49], brain tumor detection [50] and segmentation of medical image datasets [51]. However, such deep learning based methods have their own challenges. They require both quality and quantity of annotated training data, significant amount of training time, graphics processing unit computing, and can generalize poorly to a different dataset.

Both the difficulty and cost of generating annotated training data increases exponentially when dealing with Volumetric (3D) images compared with 2D, which are the desired inputs for biophysical simulations. Since the U-Net is a 2D architecture [46], it cannot be applied directly to 3D images without modifications. To this end, 3D U-net used sparsely annotated 2D slices to generate volumetric segmentations of brain tumors [52]. Similarly, VoxRestNet [53] introduced residual learning using ResNet [54], a deep residual network capable of training hundreds to thousands of layers without a performance drop, to a voxelwise representation of 3D magnetic resonance (MR) images of the brain, paving the way for scalable 3D segmentation.

Excitingly, such algorithms are being made openly accessible and easy-to-use. For example, iLastik [45, 55] and Trainable Weka Segmentation [47] are both available as plugins in software packages like ImageJ. These tools provide an interactive platform for segmentation, employing supervised classification techniques like random forests as well as unsupervised clustering such as K-means [47]. Similarly, deep learning tools such as DeepCell [56] and U-Net [46, 57] are also available in various bioimage software packages. Other stand-alone tools like the Allen Cell Structure Segmenter provide a lookup table of 20 segmentation workflows that feed into an iterative deep learning model [58]. Cloud compute based segmentation plugins like CDeep3M [59] leverage Amazon Web Services (AWS) images to provide an efficient and compute-scalable tool for both 2D and 3D biomedical images.

Generating well-organized and annotated training data continues to be the major challenge for most ML segmentation methods. Crowdsourced annotation tools like Amazon’s Mechanical Turk can be useful in this context, but are still limited by the difficulty of training naive users on tracing specific structural images. Alternatively, many ML algorithms leverage transfer learning approaches using pre-trained networks such as VGG-net [60–62], AlexNet [48], and GoogleNet [63]. In fact, popular semantic segmentation and clustering networks like Fully Convolutional Networks (FCN) [64] and DECAF [65] are themselves implemented using transfer learning approaches. Such transfer learning can also be used to generalize models trained on biological data to a different cell type or experimental condition, significantly reducing the time for training and accompanying computing resources required. More recently, label-free approaches employing a U-net variant have been applied to predict cellular structure from unlabeled brightfield images [66, 67]. These methods can serve as a platform for building low cost, scalable, and efficient segmentation of 3D cellular structure.

## 4. APPLICATIONS OF ML FOR THE GENERATION OF SYNTHETIC CELLULAR STRUCTURES

There are two main aspects involved in the development of comprehensive biophysical models—(1) what is the process being modeled? and (2) what is the geometry in which this process is being modeled? Answers to the first question are based on experimental observations and specific biology. Answering the latter is significantly more challenging because of the difficulties in—(i) obtaining accurate segmentations, (ii) discovering new structure from experiments, and (iii) simultaneously visualizing multiple structures. The use of synthetically generated geometries, which can probe different arrangements of organelles within cells could be relevant for generating biologically relevant hypotheses.

A subset of ML models, called *generative* models, deal with the task of generating new synthetic but realistic images that match the training set distribution. For our purposes, such methods are relevant in the context of generating (i) noise-free images, (ii) images representative of a different cell type, structure, or time-point, and (iii) unlikely images that represent the most unique shapes of the structure being imaged. For example, by capturing the unlikely and likely shapes in our dataset, we could generate sequences of synthetic images that transition from one shape to the next. These synthetic images can be used in biophysical simulations to generate biologically relevant hypotheses.

In recent years, there has been rapid progress in applying deep generative models to natural images, text, and even medical images. Popular classes of deep generative models like Variational Autoencoders [68], Generative Adversarial Networks [69], and Autoregressive models such as PixelRNN [70] and PixelCNN [71] have achieved state of the art performance on popular image datasets such as MNIST [72], CIFAR [73] and ImageNet [74]. Each class of models has numerous modified implementations. For example, GANs alone include models like deep convolutional GAN (DCGAN) [75], conditional GAN (cGAN) [76], StackGAN [77], InfoGAN [78], and Wasserstein GAN [79] to name a few. Each model has its own distinct set of advantages and disadvantages. GANs can produce photo-realistic images at the cost of tricky training and no dimensionality reduction. VAEs allow for both generation and inference, but their naive implementation results in less photo-realistic generative examples. Autoregressive models obtain the best log-likelihoods at the cost of poor dimensionality reduction. Importantly, all of these models are unsupervised, implying that they are not limited by manual annotation that is otherwise a common challenge to supervised learning approaches.

In cell biology, much of the work in building generative models of cellular structures has been associated with the open source CellOrganizer [80–86], which uses a Gaussian Mixture Model given reference frames like the cell and nuclear shape in order to predict organelle shape distribution. These models also have the option to be parametric (parameters such as number of objects), which reduces the complexity of the learning task, the training time and GPU computing resources required, while also allowing for exploration and analysis of the parameters and their effect on the spatial organization of cells. Aside from CellOrganizer, other recent efforts have begun to leverage deep generative models in cell biology. We now have models that can predict structure localization given cell and nuclear shape [87], extract functional relationships between fluorescently tagged proteins structures in cell images [88], learn cell features from cell morphological profiling experiments [89], and interpret gene expression levels from single-cell RNA sequencing data [90, 91].

The challenge going forward will be how best to use generative modeling given the data in hand. This will depend on the question we want to ask of the data. For example, if we are modeling processes associated with cell and nuclear shape, spherical harmonics based generative models might be more appropriate than deep learning based methods [92]. If we are interested in inpainting a missing wedge from a tomogram using a generative model, then GANs might be more appropriate [93]. Generated images can also be used as a source of training data for segmentation and classification tasks [94]. Taken together, these choices will help develop efficient end-to-end pipelines for segmentation and shape generation, and provide a platform for running biophysical simulations. Already, CellOrganizer can export spatial instances to cell simulation engines such as MCell [95] and VirtualCell [96], allowing us to simulate chemical reactions in different spatial compartments. Similar pipelines for deep generative models will need to be implemented in order to fully realize their downstream interpretations.

## 5. APPLICATIONS OF ML FOR MESHING, SIMULATION, AND DATA ANALYSIS

**FIGURE 2 |:**
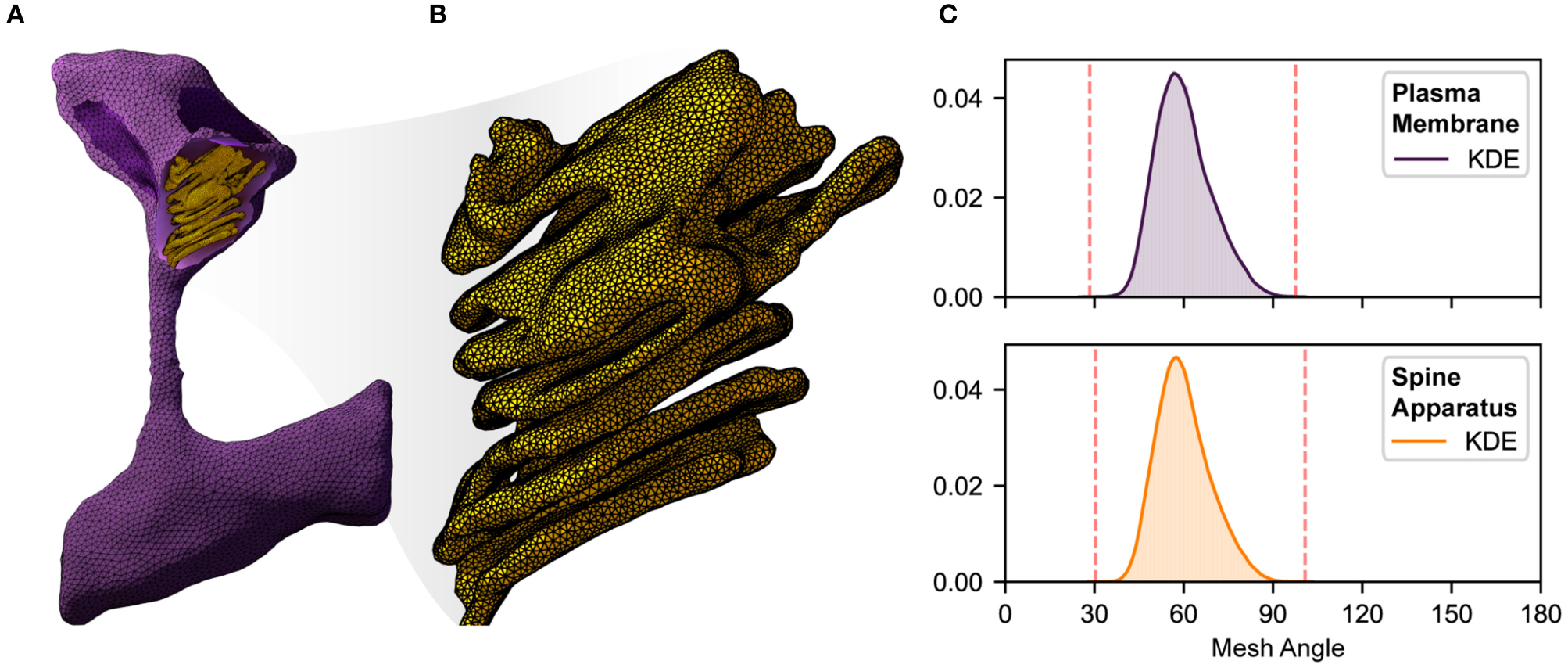
An illustration of complexity, size, quality, and local resolution of meshes typically needed for realistic simulation of biophysical systems. Meshes are generated using GAMer 2 [11, 107]. **(A)** Example surface mesh of a dendritic spine with geometry informed by electron micrographs from Wu et al. [24]. The plasma membrane is shown in purple with the post synaptic density rendered in dark purple. The spine apparatus, a specialized form of the endoplasmic reticulum is shown in yellow. **(B)** A zoomed in view of the spine apparatus. Note that the mesh density is much higher in order to represent the fine structural details. **(C)** Binned histogram distributions of mesh angles for both the plasma membrane and spine apparatus. The colored smooth lines are the result of a kernel density estimate. Dotted red lines correspond to the minimum and maximum angle values in each mesh. Both meshes are high quality with few high aspect ratio triangles (i.e., those deviating most from equilateral).

ML is commonly applied to mesh segmentation and classification; examples include PointNet [97] (segments and classifies a point cloud), and MeshCNN [98] (segments and classifies edges in a mesh). However, although the term *machine learning* was not traditionally used to describe meshing techniques, in fact algorithms for mesh generation (cf. [99]), mesh improvement (such as mesh smoothing [100]), and mesh refinement [101–104] all fundamentally involve local (cf. [105]) and/or global (cf. [106]) optimization of an *objective function* (see Figure 2). Mesh point locations, and/or edge/face connectivity decisions are viewed as parameters that are determined (or *learned*) as part of an iterative algorithm that extremizes a local or global objective function (usually involving constraints as well) in an effort to generate, improve, or refine a given mesh. In addition, *adaptive numerical methods* for simulation of physical systems involving the solution of ordinary (ODE) and partial (PDE) differential equations are again an early example of the application of ML techniques in computational science, long before the terminology was widely used. A classic reference from the 1970’s in the context of adaptive finite element methods is Babuška and Rheinboldt [108, 109]; all modern approaches to adaptive numerical methods for ODE and PDE systems continue to follow the same general framework outlined in that work: (i) Solve the ODE/PDE on the current mesh; (ii) Estimate the error using *a posteriori* indicators; (iii) Refine the mesh using provably non-degenerate local refinement with closure; (iv) Go back to step (i) and repeat the iteration until a target quality measure is obtained (a standard approach is to approximately minimize a *global error function*, through the use of local error estimates). These types of adaptive algorithms are effectively *machine learning* the best possible choice (highest accuracy with least cost) of mesh and corresponding numerical discretization for the target ODE/PDE system. Recent work in the area is now moving toward a more explicit and sophisticated use of modern ML techniques (cf. [110, 111]).

Given a high quality and high resolution mesh representation of a structural geometry (see Figure 2), we can begin to probe structure-function relationships through mathematical modeling [11, 112]. However, a single realistic geometry is not enough since it only captures a snapshot in time of the cellular geometry. Furthermore, structural variability is a hallmark of cell biology [14, 24]. Dimensionality reduction techniques like principal component analaysis (PCA) or Variational Autoencoders (VAEs) can help determine both the average and extreme representations of a distribution of shapes, providing a starting point for running simulations. Generative models (discussed in section 4) can then be used to populate a single cell with multiple learned distributions—for example, generate cell shapes from EM images overlaid with protein structure shapes learned from fluorescence images [10, 80, 87].

To facilitate population studies, it is important that structural datasets be made publicly available, as they commonly are in neuroscience [23, 24, 113]. This is following in the footsteps of - omics datasets such as genomics, proteomics and transcriptomics [114], which have traditionally been made public in large scale projects like the Cancer Genome Atlas [115], the Human Microbiome Project [116] and the ENCODE project consortium [117]. ML can then be used to identify structure-phenotype associations, in much the same way as genotype-phenotype relationships are predicted from -omics studies [118, 119].

Importantly, by running simulations on distributions of realistic shapes, we can generate experimentally testable hypotheses. This is much harder in -omics datasets, where mechanistic insight is usually obtained via constraint based modeling [119]. Further, we can also explore the implications of assuming an idealistic geometry—a common assumption in hypothesis-driven modeling. For example, idealized geometries have been the starting point of many signaling models that explore spatio-temporal dynamics using deterministic reaction-diffusion formulations [4, 5, 17, 18, 120–123] or using Brownian dynamics or other formulations [95, 124–133]. An excellent example of insights gained using these idealized geometries is in exploring how changing the diffusion distances can affect the dynamics of signaling molecules such as Ca^2+^ [123]. Other physical systems that are commonly modeled and simulated include structural mechanics [2, 7], fluid mechanics [112, 134] and thermodynamics [135].

A major bottleneck in setting up accurate computational simulations of biophysical systems, idealistic or otherwise, revolve around the choice of constitutive equations, estimation of the free parameters such as reaction rate constants, diffusion coefficients, and material properties, and computational algorithms for solving the resulting governing equations numerically on these domains. While there is a large history of mathematical modeling in biology to set the stage for constitutive equations, estimation of free parameters remains a major challenge. Another major challenge for physically realistic models of signaling is knowing the location of the various molecules involved. Realistic geometries pose the additional challenge of requiring us to first understand the distribution of shapes, followed by analyzing simulation results across that distribution. Similar to how ML can be used in adaptive numerical methods to output a good mesh, ML can also be used for adaptive nonlinear data fitting to determine biophysical parameters with uncertainity estimates [136, 137]. Incorporating domain knowledge such as stress-strain relationships [138] or statistical molecular dynamic states [139] into ML algorithms can also improve interpretability while closing the loop between ML frameworks and biophysical modeling.

## 6. PERSPECTIVES AND FUTURE DIRECTIONS

In this perspective, we have discussed three key aspects of a pipeline for realistic cellular simulations: (i) Reconstruction and segmentation of cellular structure; (ii) Generation of cellular structure; and (iii) Mesh generation, refinement and simulation. While these were discussed separately, neural networks like Pixel2Mesh demonstrate the feasibility of end-to-end pipelines from a single black box [140]. Of course, black boxes are not interpretable, and recent ML frameworks using Visible Neural Networks [141] demonstrate the potential of incorporating extensive prior knowledge to create a fully interpretable neural network capable of highlighting functional changes to every neuron/subsystem upon perturbing the input. Other ML frameworks like SAUCIE use regularizations to enforce mechanistic intepretability in the hidden layers of an autoencoder neural network [142]. We anticipate that future endeavors will implement a fully interpretable and end-to-end pipeline for biophysical simulations.

## References

[1] Lee CT, Amaro RE. Exascale computing: a new dawn for computational biology. Comput Sci Eng. (2018) 20:18–25. doi: 10.1109/MCSE.2018.05329812 30983889 PMC6458592

[2] Alimohamadi H, Vasan R, Hassinger JE, Stachowiak JC, Rangamani P. The role of traction in membrane curvature generation. Mol Biol Cell. (2018) 29:2024–35. doi: 10.1091/mbc.E18-02-0087 30044708 PMC6232966

[3] Vasan R, Akamatsu M, Schöneberg J, Rangamani P. Intracellular membrane trafficking: modeling local movements in cells. In: Stolarska M, Tarfulea N, editors. Cell Movement. Cham: Springer (2018). p. 259–301. doi: 10.1007/978-3-319-96842-1_9

[4] Bell M, Bartol T, Sejnowski T, Rangamani P. Dendritic spine geometry and spine apparatus organization govern the spatiotemporal dynamics of calcium. J Gen Physiol. (2019) 151:2221. doi: 10.1085/jgp.20181226107312019a 31324651 PMC6683673

[5] Ohadi D, Rangamani P. Geometric control of frequency modulation of cAMP oscillations due to Ca2+-bursts in dendritic spines. Biophys J. (2019) 117:1981–94. doi: 10.1101/520643 31668747 PMC7018999

[6] Vasan R, Maleckar MM, Williams CD, Rangamani P. DLITE uses cell-cell interface movement to better infer cell-cell tensions. Biophys J. (2019) 117:1714–27. doi: 10.1016/j.bpj.2019.09.034 31648791 PMC6838938

[7] Vasan R, Rudraraju S, Garikipati K, Akamatsu M, Rangamani P. A mechanical model reveals that non-axisymmetric buckling lowers the energy barrier associated with membrane neck constriction. Soft Matter. (2019). doi: 10.1101/672485 31830191

[8] Rudraraju S, Van der Ven A, Garikipati K. Mechanochemical spinodal decomposition: a phenomenological theory of phase transformations in multi-component, crystalline solids. npj Comput Mater. (2016) 2:16012. doi: 10.1038/npjcompumats.2016.12

[9] Mihai LA, Budday S, Holzapfel GA, Kuhl E, Goriely A. A family of hyperelastic models for human brain tissue. J Mech Phys Solids. (2017) 106:60–79. doi: 10.1016/j.jmps.2017.05.015

[10] Murphy RF. Building cell models and simulations from microscope images. Methods. (2016) 96:33–9. doi: 10.1016/j.ymeth.2015.10.011 26484733 PMC4766043

[11] Lee CT, Laughlin JG, Moody JB, Amaro RE, McCammon JA, Holst MJ, An open source mesh generation platform for biophysical modeling using realistic cellular geometries. arXiv:190904781. (2019). doi: 10.1101/765453 PMC7063475 32032503

[12] Rangamani P, Lipshtat A, Azeloglu EU, Calizo RC, Hu M, Ghassemi S, Decoding information in cell shape. Cell. (2013) 154:1356–69. doi: 10.1016/j.cell.2013.08.026 24034255 PMC3874130

[13] Deuling H, Helfrich W. Red blood cell shapes as explained on the basis of curvature elasticity. Biophys J. (1976) 16:861–8. doi: 10.1016/S0006-3495(76)85736-0 938726 PMC1334911

[14] Bartol TM Jr, Bromer C, Kinney J, Chirillo MA, Bourne JN, Harris KM, Nanoconnectomic upper bound on the variability of synaptic plasticity. Elife. (2015) 4:e10778. doi: 10.7554/eLife.10778 26618907 PMC4737657

[15] Ritz R, Sejnowski TJ. Synchronous oscillatory activity in sensory systems: new vistas on mechanisms. Curr Opin Neurobiol. (1997) 7:536–46. doi: 10.1016/S0959-4388(97)80034-7 9287205

[16] Harris KM, Kater S. Dendritic spines: cellular specializations imparting both stability and flexibility to synaptic function. Annu Rev Neurosci. (1994) 17:341–71. doi: 10.1146/annurev.ne.17.030194.002013 8210179

[17] Cugno A, Bartol TM, Sejnowski TJ, Iyengar R, Rangamani P. Geometric principles of second messenger dynamics in dendritic spines. Sci Rep. (2019) 9:1–18. doi: 10.1038/s41598-019-48028-0 31406140 PMC6691135

[18] Ohadi D, Schmitt DL, Calabrese B, Halpain S, Zhang J, Rangamani P. Computational modeling reveals frequency modulation of calcium-cAMP/PKA pathway in dendritic spines. Biophys J. (2019) 117:1963–80. doi: 10.1101/521740 31668749 PMC7031750

[19] Huang F, Sirinakis G, Allgeyer ES, Schroeder LK, Duim WC, Kromann EB, Ultra-high resolution 3D imaging of whole cells. Cell. (2016) 166:1028–40. doi: 10.1016/j.cell.2016.06.016 27397506 PMC5005454

[20] Graf BW, Boppart SA. Imaging and analysis of three-dimensional cell culture models. In: Papkovsky DB, editor. Live Cell Imaging. Springer (2010). p. 211–27. 10.1007/978-1-60761-404-3_13 PMC3699323 19957133

[21] Lichtman JW, Pfister H, Shavit N. The big data challenges of connectomics. Nat Neurosci. (2014) 17:1448. doi: 10.1038/nn.3837 25349911 PMC4412267

[22] Maher G, Wilson N, Marsden A. Accelerating cardiovascular model building with convolutional neural networks. Med Biol Eng Comput. (2019) 57:2319–35. doi: 10.1007/s11517-019-02029-3 31446517 PMC7250144

[23] Januszewski M, Kornfeld J, Li PH, Pope A, Blakely T, Lindsey L, High-precision automated reconstruction of neurons with flood-filling networks. Nat Methods. (2018) 15:605. doi: 10.1038/s41592-018-0049-4 30013046

[24] Wu Y, Whiteus C, Xu CS, Hayworth KJ, Weinberg RJ, Hess HF, Contacts between the endoplasmic reticulum and other membranes in neurons. Proc Natl Acad Sci USA. (2017) 114:E4859–67. doi: 10.1073/pnas.1701078114 28559323 PMC5474793

[25] van Aarle W, Palenstijn WJ, De Beenhouwer J, Altantzis T, Bals S, Batenburg KJ, The ASTRA toolbox: a platform for advanced algorithm development in electron tomography. Ultramicroscopy. (2015) 157:35–47. doi: 10.1016/j.ultramic.2015.05.002 26057688

[26] Lidke DS, Lidke KA. Advances in high-resolution imaging–techniques for three-dimensional imaging of cellular structures. J Cell Sci. (2012) 125:2571–80. doi: 10.1242/jcs.090027 22685332 PMC3706075

[27] Moen E, Bannon D, Kudo T, Graf W, Covert M, Van Valen D. Deep learning for cellular image analysis. Nat Methods. (2019) 16:1233–46. doi: 10.1038/s41592-019-0403-1 31133758 PMC8759575

[28] Koster A, Chen H, Sedat J, Agard D. Automated microscopy for electron tomography. Ultramicroscopy. (1992) 46:207–27. doi: 10.1016/0304-3991(92)90016-D 1481272

[29] Mazel T, Raymond R, Raymond-Stintz M, Jett S, Wilson BS. Stochastic modeling of calcium in 3D geometry. Biophys J. (2009) 96:1691–706. doi: 10.1016/j.bpj.2008.10.066 19254531 PMC2996128

[30] West M, Zurek N, Hoenger A, Voeltz GK. A 3D analysis of yeast ER structure reveals how ER domains are organized by membrane curvature. J Cell Biol. (2011) 193:333–46. doi: 10.1083/jcb.201011039 21502358 PMC3080256

[31] Noske AB, Costin AJ, Morgan GP, Marsh BJ. Expedited approaches to whole cell electron tomography and organelle mark-up *in situ* in high-pressure frozen pancreatic islets. J Struct Biol. (2008) 161:298–313. doi: 10.1016/j.jsb.2007.09.015 18069000 PMC2396228

[32] Perkins G, Renken C, Martone M, Young S, Ellisman M, Frey T. Electron tomography of neuronal mitochondria: three-dimensional structure and organization of cristae and membrane contacts. J Struct Biol. (1997) 119:260–72. doi: 10.1006/jsbi.1997.3885 9245766

[33] Baker LA, Rubinstein JL. Radiation damage in electron cryomicroscopy. In: Jensen GJ, editor. Methods in Enzymology. vol. 481. Elsevier (2010). p. 371–88. doi: 10.1016/S0076-6879(10)81015-8 20887865

[34] Phan S, Boassa D, Nguyen P, Wan X, Lanman J, Lawrence A, 3D reconstruction of biological structures: automated procedures for alignment and reconstruction of multiple tilt series in electron tomography. Adv Struct Chem Imaging. (2017) 2:8. doi: 10.1186/s40679-016-0021-2 27547706 PMC4972035

[35] Kremer JR, Mastronarde DN, McIntosh JR. Computer visualization of three-dimensional image data using IMOD. J Struct Biol. (1996) 116:71–6. doi: 10.1006/jsbi.1996.0013 8742726

[36] MessaoudiI C, Boudier T, Sorzano COS, Marco S. TomoJ: tomography software for three-dimensional reconstruction in transmission electron microscopy. BMC Bioinformatics. (2007) 8:288. doi: 10.1186/1471-2105-8-288 17683598 PMC1976622

[37] Leary R, Saghi Z, Midgley PA, Holland DJ. Compressed sensing electron tomography. Ultramicroscopy. (2013) 131:70–91. doi: 10.1016/j.ultramic.2013.03.019 23834932

[38] Yin X, Kidd GJ, Ohno N, Perkins GA, Ellisman MH, Bastian C, Proteolipid protein–deficient myelin promotes axonal mitochondrial dysfunction via altered metabolic coupling. J Cell Biol. (2016) 215:531–42. doi: 10.1083/jcb.201607099 27872255 PMC5119941

[39] Le Borgne H, Guyader N, Guérin-Dugué A, Hérault J. Classification of images: ICA filters vs human perception. In: Proceedings of the Seventh International Symposium on Signal Processing and Its Applications, 2003. vol. 2. Paris: IEEE (2003). p. 251–4.

[40] Abràmoff MD, Magalhães PJ, Ram SJ. Image processing with ImageJ. Biophoton Int. (2004) 11:36–42.

[41] Jerman T, Pernuš F, Likar B, Špiclin Ž. Enhancement of vascular structures in 3D and 2D angiographic images. IEEE Trans Med Imaging. (2016) 35:2107–118. doi: 10.1109/TMI.2016.2550102 27076353

[42] Frangakis AS, Hegerl R. Noise reduction in electron tomographic reconstructions using nonlinear anisotropic diffusion. J Struct Biol. (2001) 135:239–50. doi: 10.1006/jsbi.2001.4406 11722164

[43] Arganda-Carreras I, Turaga SC, Berger DR, Cireşan D, Giusti A, Gambardella LM, Crowdsourcing the creation of image segmentation algorithms for connectomics. Front Neuroanat. (2015) 9:142. doi: 10.3389/fnana.2015.00142 26594156 PMC4633678

[44] Maška M, Ulman V, Svoboda D, Matula P, Matula P, Ederra C, A benchmark for comparison of cell tracking algorithms. Bioinformatics. (2014) 30:1609–17. doi: 10.1093/bioinformatics/btu080 24526711 PMC4029039

[45] Sommer C, Straehle C, Koethe U, Hamprecht FA. Ilastik: interactive learning and segmentation toolkit. In: 2011 IEEE International Symposium on Biomedical Imaging: From Nano to Macro. Chicago, IL: IEEE (2011). p. 230–3. doi: 10.1109/ISBI.2011.5872394

[46] Ronneberger O, Fischer P, Brox T. U-net: convolutional networks for biomedical image segmentation. In: International Conference on Medical Image Computing and Computer-Assisted Intervention. Cham: Springer (2015). p. 234–41.

[47] Arganda-Carreras I, Kaynig V, Rueden C, Eliceiri KW, Schindelin J, Cardona A, Trainable Weka Segmentation: a machine learning tool for microscopy pixel classification. Bioinformatics. (2017) 33:2424–6. doi: 10.1093/bioinformatics/btx180 28369169

[48] Krizhevsky A, Sutskever I, Hinton GE. Imagenet classification with deep convolutional neural networks. In: Advances in Neural Information Processing Systems (2012). p. 1097–105.

[49] Poplin R, Chang PC, Alexander D, Schwartz S, Colthurst T, Ku A, A universal SNP and small-indel variant caller using deep neural networks. Nat Biotechnol. (2018) 36:983. doi: 10.1038/nbt.4235 30247488

[50] Dong H, Yang G, Liu F, Mo Y, Guo Y. Automatic brain tumor detection and segmentation using U-Net based fully convolutional networks. In: Annual Conference on Medical Image Understanding and Analysis. Cham: Springer (2017). p. 506–17.

[51] Weng Y, Zhou T, Li Y, Qiu X. NAS-Unet: neural architecture search for medical image segmentation. IEEE Access. (2019) 7:44247–57. doi: 10.1109/ACCESS.2019.2908991

[52] Çiçek Ö, Abdulkadir A, Lienkamp SS, Brox T, Ronneberger O. 3D U-Net: learning dense volumetric segmentation from sparse annotation. In: International Conference on Medical Image Computing and Computer-Assisted Intervention. Cham: Springer (2016). p. 424–32.

[53] Chen H, Dou Q, Yu L, Qin J, Heng PA. VoxResNet: deep voxelwise residual networks for brain segmentation from 3D MR images. NeuroImage. (2018) 170:446–55. doi: 10.1016/j.neuroimage.2017.04.041 28445774

[54] He K, Zhang X, Ren S, Sun J. Identity mappings in deep residual networks. In: European Conference on Computer Vision. Cham: Springer (2016). p. 630–45.

[55] Berg S, Kutra D, Kroeger T, Straehle CN, Kausler BX, Haubold C, ilastik: interactive machine learning for (bio) image analysis. Nat Methods. (2019) 16:1–7. doi: 10.1038/s41592-019-0582-9 31570887

[56] Van Valen DA, Kudo T, Lane KM, Macklin DN, Quach NT, DeFelice MM, Deep learning automates the quantitative analysis of individual cells in live-cell imaging experiments. PLoS Comput Biol. (2016) 12:e1005177. doi: 10.1371/journal.pcbi.1005177 27814364 PMC5096676

[57] Falk T, Mai D, Bensch R, Çiçek Ö, Abdulkadir A, Marrakchi Y, U-Net: deep learning for cell counting, detection, and morphometry. Nat Methods. (2019) 16:67. doi: 10.1038/s41592-018-0261-2 30559429

[58] Chen J, Ding L, Viana MP, Hendershott MC, Yang R, Mueller IA, The Allen Cell Structure Segmenter: a new open source toolkit for segmenting 3D intracellular structures in fluorescence microscopy images. bioRxiv. (2018) 491035. doi: 10.1101/491035

[59] Haberl MG, Churas C, Tindall L, Boassa D, Phan S, Bushong EA, CDeep3M—Plug-and-Play cloud-based deep learning for image segmentation. Nat Methods. (2018) 15:677. doi: 10.1038/s41592-018-0106-z 30171236 PMC6548193

[60] Bruna J, Sprechmann P, LeCun Y. Super-resolution with deep convolutional sufficient statistics. arXiv:151105666. (2015).

[61] Johnson J, Alahi A, Fei-Fei L. Perceptual losses for real-time style transfer and super-resolution. In: European Conference on Computer Vision. Cham: Springer (2016). p. 694–711.

[62] Simonyan K, Zisserman A. Very deep convolutional networks for large-scale image recognition. arXiv 1409.1556 (2014).

[63] Szegedy C, Liu W, Jia Y, Sermanet P, Reed S, Anguelov D, Going deeper with convolutions. In: Proceedings of the IEEE Conference on Computer Vision and Pattern Recognition (2015). p. 1–9.

[64] Long J, Shelhamer E, Darrell T. Fully convolutional networks for semantic segmentation. In: Proceedings of the IEEE Conference on Computer Vision and Pattern Recognition (2015). p. 3431–40. 10.1109/TPAMI.2016.2572683 27244717

[65] Donahue J, Jia Y, Vinyals O, Hoffman J, Zhang N, Tzeng E, Decaf: a deep convolutional activation feature for generic visual recognition. In: International Conference on Machine Learning (2014). p. 647–55.

[66] Ounkomol C, Seshamani S, Maleckar MM, Collman F, Johnson GR. Label-free prediction of three-dimensional fluorescence images from transmitted-light microscopy. Nat Methods. (2018) 15:917. doi: 10.1038/s41592-018-0111-2 30224672 PMC6212323

[67] Christiansen EM, Yang SJ, Ando DM, Javaherian A, Skibinski G, Lipnick S, *In silico* labeling: predicting fluorescent labels in unlabeled images. Cell. (2018) 173:792–803. doi: 10.1016/j.cell.2018.03.040 29656897 PMC6309178

[68] Kingma DP, Welling M. Auto-encoding variational bayes. arXiv: 13126114 (2013).

[69] Goodfellow I, Pouget-Abadie J, Mirza M, Xu B, Warde-Farley D, Ozair S, Generative adversarial nets. In: Advances in Neural Information Processing Systems (2014). p. 2672–80.

[70] Avd Oord, Kalchbrenner N, Kavukcuoglu K. Pixel recurrent neural networks. arXiv:160106759 (2016).

[71] Van den Oord A, Kalchbrenner N, Espeholt L, Vinyals O, Graves A, Kavukcuoglu K. Conditional image generation with pixelcnn decoders. In: Advances in Neural Information Processing Systems (2016). p. 4790–8.

[72] Deng L The MNIST database of handwritten digit images for machine learning research [best of the web]. IEEE Signal Process Mag. (2012) 29:141–2. doi: 10.1109/MSP.2012.2211477

[73] Krizhevsky A, Hinton G. Learning Multiple Layers of Features From Tiny Images. Vol. 1. Technical Report, University of Toronto (2009).

[74] Deng J, Dong W, Socher R, Li LJ, Li K, Fei-Fei L. Imagenet: a large-scale hierarchical image database. In: 2009 IEEE Conference on Computer Vision and Pattern Recognition. IEEE (2009). p. 248–55. doi: 10.1109/CVPR.2009.5206848

[75] Radford A, Metz L, Chintala S. Unsupervised representation learning with deep convolutional generative adversarial networks. arXiv:151106434. (2015).

[76] Antipov G, Baccouche M, Dugelay JL. Face aging with conditional generative adversarial networks. In: 2017 IEEE International Conference on Image Processing (ICIP). IEEE (2017). p. 2089–93.

[77] Zhang H, Xu T, Li H, Zhang S, Wang X, Huang X, Stackgan: text to photo-realistic image synthesis with stacked generative adversarial networks. In: Proceedings of the IEEE International Conference on Computer Vision (2017). p. 5907–15.

[78] Chen X, Duan Y, Houthooft R, Schulman J, Sutskever I, Abbeel P. Infogan: interpretable representation learning by information maximizing generative adversarial nets. In: Advances in Neural Information Processing Systems. (2016). p. 2172–80.

[79] Arjovsky M, Chintala S, Bottou L. Wasserstein gan. arXiv:170107875 (2017).

[80] Johnson GR, Buck TE, Sullivan DP, Rohde GK, Murphy RF. Joint modeling of cell and nuclear shape variation. Mol Biol Cell. (2015) 26:4046–56. doi: 10.1091/mbc.E15-06-0370 26354424 PMC4710235

[81] Johnson GR, Li J, Shariff A, Rohde GK, Murphy RF. Automated learning of subcellular variation among punctate protein patterns and a generative model of their relation to microtubules. PLoS Comput Biol. (2015) 11:e1004614. doi: 10.1371/journal.pcbi.1004614 26624011 PMC4704559

[82] Shariff A, Kangas J, Coelho LP, Quinn S, Murphy RF. Automated image analysis for high-content screening and analysis. J Biomol Screen. (2010) 15:726–34. doi: 10.1177/1087057110370894 20488979

[83] Rohde GK, Ribeiro AJ, Dahl KN, Murphy RF. Deformation-based nuclear morphometry: capturing nuclear shape variation in HeLa cells. Cytometry A. (2008) 73:341–50. doi: 10.1002/cyto.a.20506 18163487

[84] Shariff A, Murphy RF, Rohde GK. Automated estimation of microtubule model parameters from 3-d live cell microscopy images. In: 2011 IEEE International Symposium on Biomedical Imaging: From Nano to Macro. IEEE (2011). p. 1330–3. doi: 10.1109/ISBI.2011.5872646 PMC3146051 21804927

[85] Peng T, Murphy RF. Image-derived, three-dimensional generative models of cellular organization. Cytometry A. (2011) 79:383–91. doi: 10.1002/cyto.a.21066 21472848 PMC3127045

[86] Zhao T, Murphy RF. Automated learning of generative models for subcellular location: building blocks for systems biology. Cytometry A. (2007) 71:978–90. doi: 10.1002/cyto.a.20487 17972315

[87] Johnson GR, Donovan-Maiye RM, Maleckar MM. Generative modeling with conditional autoencoders: building an integrated cell. arXiv:170500092 (2017). doi: 10.1101/238378

[88] Osokin A, Chessel A, Carazo Salas RE, Vaggi F. GANs for biological image synthesis. In: Proceedings of the IEEE International Conference on Computer Vision (2017). p. 2233–42.

[89] Caicedo JC, McQuin C, Goodman A, Singh S, Carpenter AE. Weakly supervised learning of single-cell feature embeddings. In: Proceedings of the IEEE Conference on Computer Vision and Pattern Recognition (2018). p. 9309–18. doi: 10.1101/293431 PMC6432648 30918435

[90] Lopez R, Regier J, Cole MB, Jordan MI, Yosef N. Deep generative modeling for single-cell transcriptomics. Nat Methods. (2018) 15:1053. doi: 10.1038/s41592-018-0229-2 30504886 PMC6289068

[91] Ding J, Condon A, Shah SP. Interpretable dimensionality reduction of single cell transcriptome data with deep generative models. Nat Commun. (2018) 9:2002. doi: 10.1038/s41467-018-04368-5 29784946 PMC5962608

[92] Ruan X, Murphy RF. Evaluation of methods for generative modeling of cell and nuclear shape. Bioinformatics. (2018) 35:2475–85. doi: 10.1093/bioinformatics/bty983 PMC6612826 30535313

[93] Ding G, Liu Y, Zhang R, Xin HL. A joint deep learning model to recover information and reduce artifacts in missing-wedge sinograms for electron tomography and beyond. Sci Rep. (2019) 9:1–13. doi: 10.1038/s41598-019-49267-x 31488874 PMC6728317

[94] Yang D, Deng J. Learning to generate synthetic 3D training data through hybrid gradient. arXiv: 190700267 (2019).

[95] Stiles JR, Bartol TM, Monte Carlo methods for simulating realistic synaptic microphysiology using MCell. Comput Neurosci. (2001) 87–127. doi: 10.1201/9781420039290.ch4

[96] Loew LM, Schaff JC. The Virtual Cell: a software environment for computational cell biology. Trends Biotechnol. (2001) 19:401–6. doi: 10.1016/S0167-7799(01)01740-1 11587765

[97] Qi CR, Su H, Mo K, Guibas LJ. Pointnet: deep learning on point sets for 3d classification and segmentation. In: Proceedings of the IEEE Conference on Computer Vision and Pattern Recognition (2017). p. 652–60.

[98] Hanocka R, Hertz A, Fish N, Giryes R, Fleishman S, Cohen-Or D. MeshCNN: a network with an edge. ACM Trans Graph. (2019) 38:90. doi: 10.1145/3306346.3322959

[99] Lee C, Moody J, Amaro R, McCammon J, Holst M. The implementation of the colored abstract simplicial complex and its application to mesh generation. ACM Trans Math Softw. (2019) 45:28:1–20. doi: 10.1145/3321515 31474782 PMC6716611

[100] Bank RE, Smith RK. Mesh smoothing using *A Posteriori* error estimates. SIAM J Numer Anal. (1997) 34:979–97. doi: 10.1137/S0036142994265292

[101] Liu A, Joe B. Quality local refinement of tetrahedral meshes based on bisection. SIAM J Sci Stat Comput. (1995) 16:1269–91. doi: 10.1137/0916074

[102] Maubach JM. Local bisection refinement for N-simplicial grids generated by relection. SIAM J Sci Stat Comput. (1995) 16:210–77. doi: 10.1137/0916014

[103] Bey J Tetrahedral grid refinement. Computing. (1995) 55:355–78. doi: 10.1007/BF02238487

[104] Arnold DN, Mukherjee A, Pouly L. Locally adapted tetrahedral meshes using bisection. SIAM J Sci Stat Comput. (1997) 22:431–48. doi: 10.1137/S1064827597323373

[105] Gao Z, Yu Z, Holst M. Feature-preserving surface mesh smoothing via suboptimal delaunay triangulation. Graph Models. (2013) 75:23–38. doi: 10.1016/j.gmod.2012.10.007 23580890 PMC3619448

[106] Chen L, Holst M. Efficient mesh optimization schemes based on optimal delaunay triangulations. Comp Meth Appl Mech Eng. (2011) 200:967–84. doi: 10.1016/j.cma.2010.11.007

[107] Lee C, Laughlin J, Angliviel de La Beaumelle N, Amaro R, McCammon JA, Ramamoorthi R, GAMer 2: a system for 3D mesh processing of cellular electron micrographs (2019). arXiv:1901.11008 [q-bio.QM]. doi: 10.1101/534479 PMC7162555 32251448

[108] Babuška I, Rheinboldt WC. A posteriori error estimates for the finite element method. Int J Numer Methods Eng. (1978) 12:1597–615. doi: 10.1002/nme.1620121010

[109] Babuška I, Rheinboldt WC. Error estimates for adaptive finite element computations. SIAM J Numer Anal. (1978) 15:736–54. doi: 10.1137/0715049

[110] Fritzen F, Fernández M, Larsson F. On-the-fly adaptivity for nonlinear twoscale simulations using artificial neural networks and reduced order modeling. Front Mater. (2019) 6:75. doi: 10.3389/fmats.2019.00075

[111] Manevitz LM, Bitar A, Givoli D. Neural network time series forecasting of finite-element mesh adaptation. Neurocomputing. (2005) 63:447–63. doi: 10.1016/j.neucom.2004.06.009

[112] Updegrove A, Wilson NM, Merkow J, Lan H, Marsden AL, Shadden SC. SimVascular: an open source pipeline for cardiovascular simulation. Ann Biomed Eng. (2017) 45:525–41. doi: 10.1007/s10439-016-1762-8 27933407 PMC6546171

[113] Kasthuri N, Hayworth KJ, Berger DR, Schalek RL, Conchello JA, Knowles-Barley S, Saturated reconstruction of a volume of neocortex. Cell. (2015) 162:648–61. doi: 10.1016/j.cell.2015.06.054 26232230

[114] Perez-Riverol Y, Zorin A, Dass G, Vu MT, Xu P, Glont M, Quantifying the impact of public omics data. Nat Commun. (2019) 10:1–10. doi: 10.1038/s41467-019-11461-w 31383865 PMC6683138

[115] Weinstein JN, Collisson EA, Mills GB, Shaw KRM, Ozenberger BA, Ellrott K, The cancer genome atlas pan-cancer analysis project. Nat Genet. (2013) 45:1113. doi: 10.1038/ng.2764 24071849 PMC3919969

[116] Turnbaugh PJ, Ley RE, Hamady M, Fraser-Liggett CM, Knight R, Gordon JI. The human microbiome project. Nature. (2007) 449:804. doi: 10.1038/nature06244 17943116 PMC3709439

[117] Maher B ENCODE: the human encyclopaedia. Nature News. (2012) 489:46. doi: 10.1038/489046a 22962707

[118] Camacho DM, Collins KM, Powers RK, Costello JC, Collins JJ. Next-generation machine learning for biological networks. Cell. (2018) 173:1581–92. doi: 10.1016/j.cell.2018.05.015 29887378

[119] Zampieri G, Vijayakumar S, Yaneske E, Angione C. Machine and deep learning meet genome-scale metabolic modeling. PLoS Comput Biol. (2019) 15:e1007084. doi: 10.1371/journal.pcbi.1007084 31295267 PMC6622478

[120] Resasco DC, Gao F, Morgan F, Novak IL, Schaff JC, Slepchenko BM. Virtual cell: computational tools for modeling in cell biology. WIREs Syst Biol Med. (2012) 4:129–40. doi: 10.1002/wsbm.165 PMC3288182 22139996

[121] Louch WE, Hake J, Jølle GF, Mørk HK, Sjaastad I, Lines GT, Control of Ca^2+^ release by action potential configuration in normal and failing murine cardiomyocytes. Biophys J. (2010) 99:1377–86. doi: 10.1016/j.bpj.2010.06.055 20816049 PMC2931738

[122] Yang PC, Boras BW, Jeng MT, Docken SS, Lewis TJ, McCulloch AD, A computational modeling and simulation approach to investigate mechanisms of subcellular cAMP compartmentation. PLoS Comput Biol. (2016) 12:e1005005. doi: 10.1371/journal.pcbi.1005005 27409243 PMC4943723

[123] Cheng Y, Yu Z, Hoshijima M, Holst MJ, McCulloch AD, McCammon JA, Numerical analysis of Ca2+ signaling in rat ventricular myocytes with realistic transverse-axial tubular geometry and inhibited sarcoplasmic reticulum. PLoS Comput Biol. (2010) 6:e1000972. doi: 10.1371/journal.pcbi.1000972 21060856 PMC2965743

[124] Stiles JR, Van Helden D, Bartol TM, Salpeter EE, Salpeter MM. Miniature endplate current rise times less than 100 microseconds from improved dual recordings can be modeled with passive acetylcholine diffusion from a synaptic vesicle. Proc Natl Acad Sci USA. (1996) 93:5747–52. doi: 10.1073/pnas.93.12.5747 8650164 PMC39132

[125] Kerr RA, Bartol TM, Kaminsky B, Dittrich M, Chang JCJ, Baden SB, Fast Monte Carlo simulation methods for biological reaction-diffusion systems in solution and on surfaces. SIAM J Sci Comput. (2008) 30:3126–49. doi: 10.1137/070692017 20151023 PMC2819163

[126] Chen W, De Schutter E. Parallel STEPS: large scale stochastic spatial reaction-diffusion simulation with high performance computers. Front Neuroinform. (2017) 11:13. doi: 10.3389/fninf.2017.00013 28239346 PMC5301017

[127] Hepburn I, Chen W, Wils S, De Schutter E. STEPS: efficient simulation of stochastic reaction–diffusion models in realistic morphologies. BMC Syst Biol. (2012) 6:36. doi: 10.1186/1752-0509-6-36 22574658 PMC3472240

[128] Hepburn I, Chen W, De Schutter E. Accurate reaction-diffusion operator splitting on tetrahedral meshes for parallel stochastic molecular simulations. J Chem Phys. (2016) 145:054118. doi: 10.1063/1.4960034 27497550

[129] Drawert B, Engblom S, Hellander A. URDME: a modular framework for stochastic simulation of reaction-transport processes in complex geometries. BMC Syst Biol. (2012) 6:76. doi: 10.1186/1752-0509-6-76 22727185 PMC3439286

[130] Roberts E, Stone JE, Luthey-Schulten Z. Lattice microbes: high-performance stochastic simulation method for the reaction-diffusion master equation. J Comput Chem. (2013) 34:245–55. doi: 10.1002/jcc.23130 23007888 PMC3762454

[131] Hattne J, Fange D, Elf J. Stochastic reaction-diffusion simulation with MesoRD. Bioinformatics. (2005) 21:2923–4. doi: 10.1093/bioinformatics/bti431 15817692

[132] Andrews SS, Bray D. Stochastic simulation of chemical reactions with spatial resolution and single molecule detail. Phys Biol. (2004) 1:137–51. doi: 10.1088/1478-3967/1/3/001 16204833

[133] Oliveira RF, Terrin A, Benedetto GD, Cannon RC, Koh W, Kim M, The role of type 4 phosphodiesterases in generating microdomains of cAMP: large scale stochastic simulations. PLoS ONE. (2010) 5:e11725. doi: 10.1371/journal.pone.0011725 20661441 PMC2908681

[134] Wang W, Diacovo TG, Chen J, Freund JB, King MR. Simulation of platelet, thrombus and erythrocyte hydrodynamic interactions in a 3D arteriole with *in vivo* comparison. PLoS ONE. (2013) 8:e76949. doi: 10.1371/journal.pone.0076949 24098571 PMC3788741

[135] Peskin CS, Odell GM, Oster GF. Cellular motions and thermal fluctuations: the Brownian ratchet. Biophys J. (1993) 65:316–24. doi: 10.1016/S0006-3495(93)81035-X 8369439 PMC1225726

[136] Verrelst J, Muñoz J, Alonso L, Delegido J, Rivera JP, Camps-Valls G, Machine learning regression algorithms for biophysical parameter retrieval: Opportunities for Sentinel-2 and-3. Remote Sens Environ. (2012) 118:127–39. doi: 10.1016/j.rse.2011.11.002

[137] Huys QJ, Paninski L. Smoothing of, and parameter estimation from, noisy biophysical recordings. PLoS Comput Biol. (2009) 5:e1000379. doi: 10.1371/journal.pcbi.1000379 19424506 PMC2676511

[138] Mendizabal A, Márquez-Neila P, Cotin S. Simulation of hyperelastic materials in real-time using deep learning. arXiv: 190406197 (2019). doi: 10.1016/j.media.2019.101569 31704451

[139] Noé F, Olsson S, Köhler J, Wu H. Boltzmann generators: sampling equilibrium states of many-body systems with deep learning. Science. (2019) 365:eaaw1147. doi: 10.1126/science.aaw1147 31488660

[140] Wang N, Zhang Y, Li Z, Fu Y, Liu W, Jiang YG. Pixel2mesh: generating 3d mesh models from single rgb images. In: Proceedings of the European Conference on Computer Vision (ECCV) (2018). p. 52–67.

[141] Ma J, Yu MK, Fong S, Ono K, Sage E, Demchak B, Using deep learning to model the hierarchical structure and function of a cell. Nat Methods. (2018) 15:290. doi: 10.1038/nmeth.4627 29505029 PMC5882547

[142] Amodio M, Van Dijk D, Srinivasan K, Chen WS, Mohsen H, Moon KR, Exploring single-cell data with deep multitasking neural networks. Nat Methods. (2019) 16:1139–45. doi: 10.1038/s41592-019-0576-7 31591579 PMC10164410

